# Pooled Protein Immunization for Identification of Cell Surface Antigens in *Streptococcus sanguinis*

**DOI:** 10.1371/journal.pone.0011666

**Published:** 2010-07-26

**Authors:** Xiuchun Ge, Todd Kitten, Cindy L. Munro, Daniel H. Conrad, Ping Xu

**Affiliations:** 1 Philips Institute of Oral and Craniofacial Molecular Biology, Virginia Commonwealth University, Richmond, Virginia, United States of America; 2 Center for the Study of Biological Complexity, Virginia Commonwealth University, Richmond, Virginia, United States of America; 3 Department of Microbiology and Immunology, Virginia Commonwealth University, Richmond, Virginia, United States of America; 4 Department of Adult Health Nursing, Virginia Commonwealth University, Richmond, Virginia, United States of America; University of Medicine & Dentistry of New Jersey-New Jersey Medical School, United States of America

## Abstract

**Background:**

Available bacterial genomes provide opportunities for screening vaccines by reverse vaccinology. Efficient identification of surface antigens is required to reduce time and animal cost in this technology. We developed an approach to identify surface antigens rapidly in *Streptococcus sanguinis*, a common infective endocarditis causative species.

**Methods and Findings:**

We applied bioinformatics for antigen prediction and pooled antigens for immunization. Forty-seven surface-exposed proteins including 28 lipoproteins and 19 cell wall-anchored proteins were chosen based on computer algorithms and comparative genomic analyses. Eight proteins among these candidates and 2 other proteins were pooled together to immunize rabbits. The antiserum reacted strongly with each protein and with *S. sanguinis* whole cells. Affinity chromatography was used to purify the antibodies to 9 of the antigen pool components. Competitive ELISA and FACS results indicated that these 9 proteins were exposed on *S. sanguinis* cell surfaces. The purified antibodies had demonstrable opsonic activity.

**Conclusions:**

The results indicate that immunization with pooled proteins, in combination with affinity purification, and comprehensive immunological assays may facilitate cell surface antigen identification to combat infectious diseases.

## Introduction

With antibiotic resistance increasing rapidly among bacterial pathogens because of antibiotic overuse, more attention has been paid to vaccine identification against bacterial diseases. Most successful vaccines against bacterial pathogens are those in which an immune response against a toxin (e.g. tetanus) is elicited. While pathogen attenuation has had much success with viruses, it is rare to see an attenuated version of a bacterial or parasitic pathogen used as a vaccinogen. Thus, the choice is generally limited to immunization with killed pathogens, where success rates have been quite variable [1] and side effects may be a concern. As the genomes of many pathogenic bacteria have become available, a new systematic approach for identification of vaccine candidates, termed reverse vaccinology [2], [3] has been developed. This approach brings conventional subunit vaccines into the modern era based on genome sequences. The process begins with the identification of all putative surface proteins, which are a logical choice for vaccine candidates. The surface proteins can be predicted from genomic sequences using computer programs based on signal peptides, LPXTG motifs, transmembrane helices and other surface protein prediction algorithms. The candidate genes are then cloned and the proteins expressed in *E. coli*. The immune responses of animals to the proteins are determined after injection of the purified proteins. This approach was first successfully applied to identify vaccine candidates in *Neisseria meningitidis* [2] and has since been applied to identify vaccine candidates for other bacterial pathogens including *Streptococcus pneumoniae* [4], *Streptococcus agalactiae* [5], *Staphylococcus aureus* [6], *Porphyromonas gingivalis* [7], *Chlamydia pneumoniae* [8], *Bacillus anthracis* [9], *Streptococcus suis* [10] and *Echinococcus granulosus* [11].

Although several vaccines have been identified by reverse vaccinology, there are two bottlenecks in this approach: (1) the expression of a large number of proteins is technically difficult and time-consuming; and (2) the examination of each protein in animal models is expensive and laborious. To overcome these problems, we developed an approach to identify candidate proteins by comparative genomics, to use an antigen pooling approach for the immunization regimen and to examine antigenicities of specific candidates in *Streptococcus sanguinis*. *S. sanguinis* is an indigenous and important opportunistic gram-positive human oral bacterium that has long been recognized as one of the principal causative agents of infective endocarditis (IE) - a serious heart disease [12]. We focused our search on both lipoproteins (Lpp) and cell wall-anchored (CWA) proteins that we believed were most likely to be effective vaccines via comparative genomics [13].This both decreases the amount of effort required with respect to PCR amplification, gene cloning and protein expression and gives a greater chance of success. By combination of the comparative genomics analysis and pooled antigen approach described, several surface-exposed proteins with strong antigenicity were identified in *S. sanguinis*. It should be noted that there are many bacterial and parasitic pathogens in which no successful vaccine strategy is available. Thus, this approach may be applied for evaluating antigens in the identification of subunit vaccines for other important clinical pathogens.

## Results

### Criteria for selection of vaccine candidates

To identify potential vaccine candidates, we compared surface-exposed proteins with literature-reported vaccines. We first used the *S. sanguinis* genome sequence to predict all putative proteins containing signal peptide sequences by SignalP [14]. Over 360 ORFs with signal peptides were identified. We also predicted transmembrane proteins by TMHMM [15]. There were over 600 ORFs predicted by TMHMM to possess transmembrane domains. The surface proteins were then compared with reported vaccines in streptococci. We found that two classes of proteins, Lpp and CWA, were frequently found in successful vaccines. For example, Wizeman et al [4] examined 108 putative surface proteins in *S. pneumoniae*, and six were protective in mice. Two of these proteins were CWA proteins and two were Lpp. To study Lpp vaccinogens, Lei et al [16] immunized mice with 16 *S. pyogenes* Lpp, and five produced antisera with growth-inhibitory activity against *S. pyogenes*. Numerous studies examining individual Lpp proteins have also identified these proteins as effective vaccinogens [17]–[27]. The hypothesis that Lpp and CWA proteins are useful as vaccine candidates is further supported by Maione et al [5] who examined 312 putative surface proteins of *S. agalactiae* for vaccine efficacy in mice. Three of four final protective proteins in this study were CWA proteins.

We therefore performed further bioinformatic analyses to identify all putative *S. sanguinis* ORFs belonging to both classes (see Materials and Methods, and [28], [29]). We identified 60 Lpp and 38 CWA in the *S. sanguinis* genome. Additionally, all putative Lpp and CWA proteins were examined for the number of transmembrane domains using the TMHMM program [15]. Proteins with more than two transmembrane domains were removed from the list. Not only are proteins with a high number of hydrophobic transmembrane domains very difficult to express in *E. coli* [30], they are also more likely to be misclassified membrane proteins buried beneath the gram-positive cell wall. We next eliminated from this list those proteins that were not conserved among streptococcal genomes. The rationale for this decision is that conserved proteins are more likely to possess important biological functions that prevent their antigenic variation. In addition, several streptococcal species are known to cause IE [31]. We would want a polyvalent vaccine to have broad protection against different streptococcal species [32]; thus, such conservation is desirable. We identified conserved proteins by comparative genomic analyses. Forty-seven conserved proteins including 28 Lpp and 19 CWA were identified based on the presence of orthologs in at least two other streptococcal species (Table 1). We found that about half of the listed proteins were homologs of leading vaccine candidates in other streptococci (Table 1) including 8 of the 19 CWA candidates and 16 of 28 Lpp candidates.

**Table 1:** Conserved Lpp and CWA proteins in *S. sanguinis* SK36.

ID^a^	SSA#	Lpp	CWA	VH^b^	Description
**1**	SSA_0138	+		+	Metal-binding (Zn)
2	SSA_0588	+		+	L-cystine ABC transporter, substrate-binding component
3	SSA_0260	+		+	Manganese/Zinc ABC transporter substrate-binding protein
4	SSA_1340	+		+	Zn/Mn ABC-type porter lipoprotein
5	SSA_0941	+		+	ABC-type phosphate transport system
6	SSA_1742	+		+	Ferrichrome-binding protein
7	SSA_1990	+		+	Zn-porter lipoprotein
**8**	SSA_1038	+			Lipoprotein
9	SSA_0375	+			D-methionine-binding lipoprotein (ABC-type transporter)
10	SSA_1298	+			Maltose/maltodextrin ABC transporter, sugar-binding protein
11	SSA_2165	+		+	ABC-type oligopeptide transporter, periplasmic component
12	SSA_1066	+		+	ABC-type oligopeptide transport system
13	SSA_1948	+		+	Oligopeptide-binding lipoprotein precursor
14	SSA_1949	+		+	AliA protein
15	SSA_1950	+		+	ABC-type oligopeptide transport system, periplasmic component
16	SSA_1729	+			ABC transporter substrate-binding protein-branched chain amino acid transport
17	SSA_1277	+			D-alanyl-D-alanine carboxypeptidase
18	SSA_2139	+			Membrane protein (preprotein translocase) oxaA 1 precursor
19	SSA_1629	+			Peptidyl-prolyl cis-trans isomerase, cyclophilin-type
**20**	SSA_0893	+			ATP-binding cassette transporter-like protein
**21**	SSA_0753	+		+	Foldase protein prsA precursor
22	SSA_2196	+			Hypothetical protein
**23**	SSA_0074	+			ABC transporter substrate-binding protein-sugar transport
24	SSA_1122	+		+	Thioredoxin family protein
25	SSA_1117	+		+	Hypothetical protein
26	SSA_1581	+		+	Metal-binding ABC transporter
27	SSA_1003	+			ABC transporter substrate-binding protein-multiple sugars
**28**	SSA_2352	+			ABC-type nitrate/sulfonate/bicarbonate transporter
29	SSA_0956		+	+	Surface protein D
30	SSA_0904		+		CshA-like fibrillar surface protein A
**31**	SSA_1663		+	+	Collagen-binding protein A
32	SSA_0565		+		Hypothetical protein
33	SSA_1112		+		Cell wall surface anchor family protein
34	SSA_1633		+	+	FimA fimbrial subunit-like protein
35	SSA_1632		+	+	Surface protein
36	SSA_0905		+	+	CshA-like fibrillar surface protein B
37	SSA_1634		+	+	Heme utilization/adhesion exoprotein
38	SSA_0303		+		Surface protein C
39	SSA_2023		+		Fructan beta-fructosidase precursor
40	SSA_0453		+		Type II secretory pathway, pullulanase PulA glycosidase
41	SSA_1234		+		5′-nucleotidase
42	SSA_0243		+		2′,3′-cyclic nucleotide 2′-phosphodiesterase/3′-nucleotidase bifunctional periplasmic precursor protein
43	SSA_1591		+		Dipeptidase
**44**	SSA_0146		+	+	DNA repair ATPase
45	SSA_1666		+	+	Collagen-binding surface protein
46	SSA_1019		+		Collagen-binding surface protein
47	SSA_0805		+		Collagen-binding surface protein

a ID, identifying number (numbers in bold indicate pool proteins).

b streptococcal vaccine homolog.

### Immunization with pooled proteins

**Figure 1:**
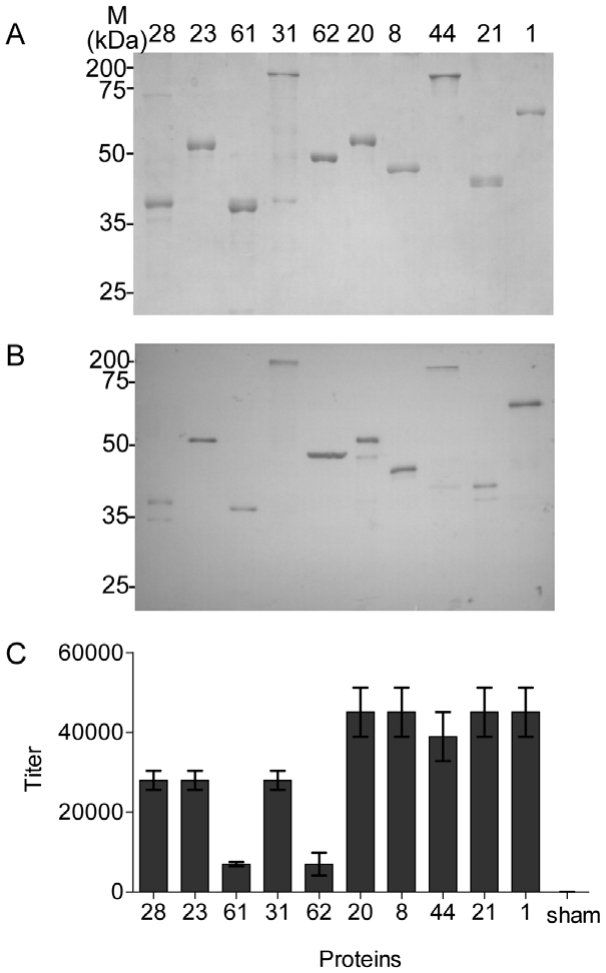
Characterization of the antibody response to pooled antigens. A. Examination of the purified, His-tagged recombinant proteins indicated above each lane by SDS-PAGE. B. Western blot of the antigens shown in A reacted with anti-pool antiserum. C. ELISA of the antigens shown in A reacted with anti-pool antiserum. The ELISA was performed in triplicate, and the mean values and standard deviations are shown.

The two most common animal models of endocarditis are rabbit [33] and rat [34], both of which have been employed by our group [35]. Both models require a relatively complicated procedure to establish IE, including insertion of a catheter through the carotid artery to the aortic valve, intravenous inoculation, and recovery of bacteria from vegetations on the aortic valve [35]. The complexity of the model precludes the individual screening of a large number of candidates. To reduce the number of animals required, we pooled candidate proteins together for immunization. To examine whether this strategy worked, we randomly picked eight protein candidates from Lpp and CWA to pool together. To compare the immunogenicity of other surface proteins, one putative secreted protein 61 and one putative surface-associated protein 62 based on signal peptide sequences predicted by SignalP 3.0 and TMHMM were put into the pool. The 10 proteins were used to immunize rabbits. Ten proteins were expressed in *E. coli* as described in Materials and Methods. To facilitate solubility and purification of the protein in *E. coli*, the region encoding hydrophobic transmembrane domains predicted by TMHMM in each ORF were excluded. After purification (Fig. 1A), equimolar amounts of each protein (total 1mg) were pooled to immunize and boost rabbits (see Materials and Methods). An equal amount (1 mg) of recombinant His-tagged *P. gingivalis* Kgp protein [25] was used for sham immunization.

To examine the reactivity of each component of the pool in immunization, a Western blot of antiserum against each protein was performed. The result showed that all ten proteins in the pool were immunogenic in rabbits (Fig. 1B). There was no reactivity of the immune serum to the recombinant sham antigen nor was there any reactivity of any of the proteins to the pre-immune serum (data not shown).

We used indirect enzyme-linked immunosorbent assay (ELISA) to detect the titer of the antiserum against specific antigens. The optimal concentrations of the reagents for ELISA were first examined by a criss-cross serial dilution with two-fold dilutions of antigen and anti-sera. The results indicated that the optimal concentration of the antigens on ELISA plates was 1–2 µg/ml. Therefore, 2 µg/ml of each antigen was used to coat plate wells in ELISA examination of the titer of immune sera. The pre-immune serum was used as the negative control. The results showed the antisera produced by pool immunization had greater reactivity against each purified antigen than the sham in the ELISA (Fig. 1C). Proteins 1, 8, 20, 21, and 44 elicited the highest immune responses in rabbits. Proteins 61 and 62 elicited lower reactivity.

We also examined surface reactivity of the antiserum using whole *S. sanguinis*-cell ELISA as described [36]. The whole-cell ELISA titers of pre-immune serum, 21-d antiserum and 42-d antiserum were 0, 128 and 32768, respectively. Thus, strong reactivity was observed at 42 days post-immunization (i.e., 21 days after boost immunization). However, there was no appreciable reactivity to surface proteins in the pool-immunized rabbits prior to vaccination and only slight reactivity at the time of the boost. These data strongly implied surface expression of at least some of the pooled proteins.

### Isolation of antigen-specific antibody by affinity purification

**Figure 2:**
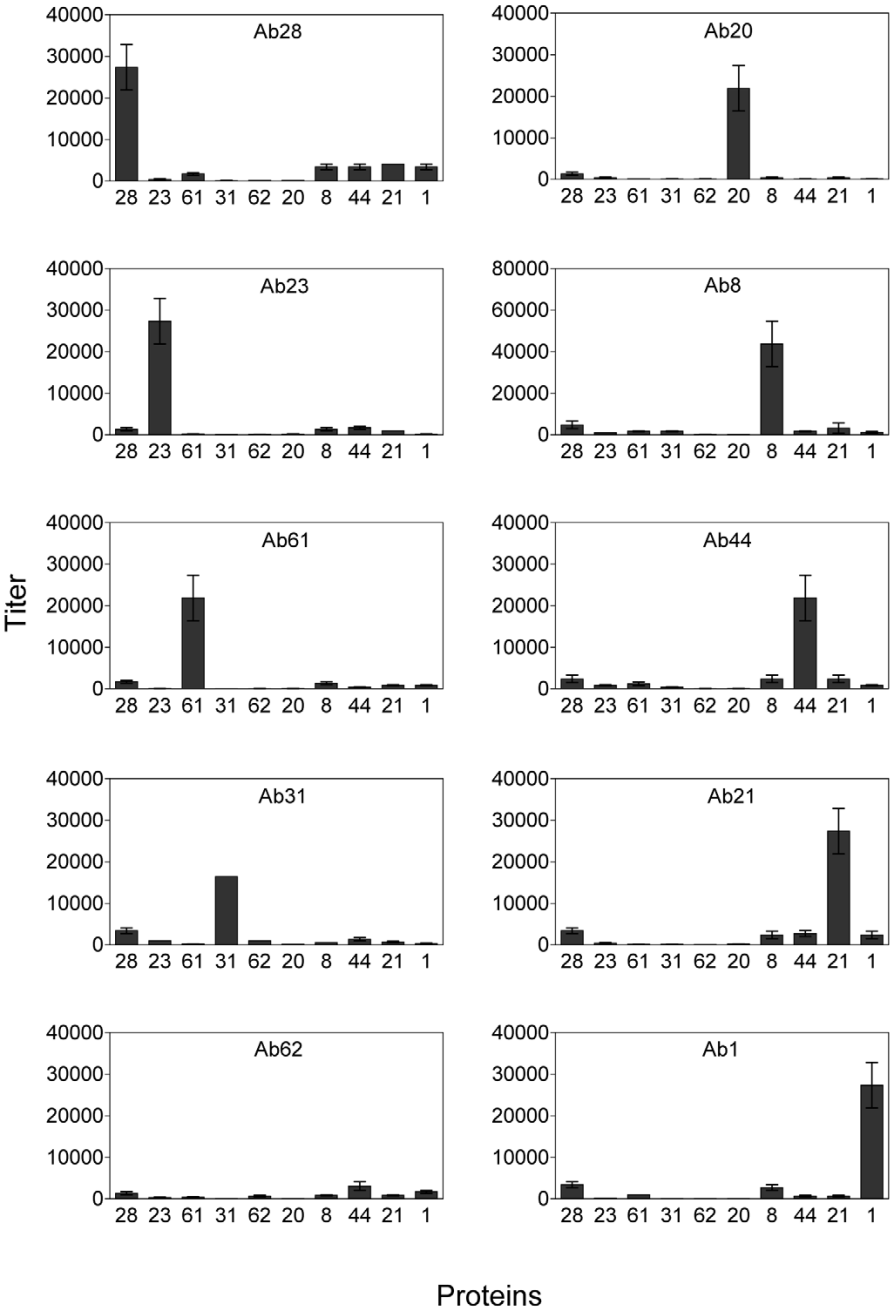
ELISA of affinity-purified antibodies against component pool antigens. Two-fold serial dilutions of affinity-purified antibodies were added to microtiter plate wells coated with individual proteins. Assays were performed in triplicate, and the mean values and standard deviations are shown. Values for cognate antigens were significantly greater than the values for all non-cognate antigens (P<0.05) except Ab62.

The eventual goal of reverse vaccinology is to identify individual vaccine candidates. The pool approach above significantly increased the efficiency of vaccine screening. However, it produced an antiserum with activities against all component proteins. In order to determine the relative importance of individual pool components, we established an affinity chromatographic method to purify antibodies from the antiserum (see Materials and Methods). Each antibody was affinity purified by a specific column covalently linked with a purified protein. The concentration of each purified antibody was determined by OD_280_. The antibodies were adjusted to equal values and the specific titer, specificity, and purity of each purified antibody was determined using ELISA (Fig. 2). The results suggested that the affinity-purified antibodies were successfully separated using our method. Except for Ab 62, each of the affinity-purified antibodies showed high titers against their specific antigens and very low titers against other proteins.

### Cell surface exposure of candidate proteins

**Figure 3:**
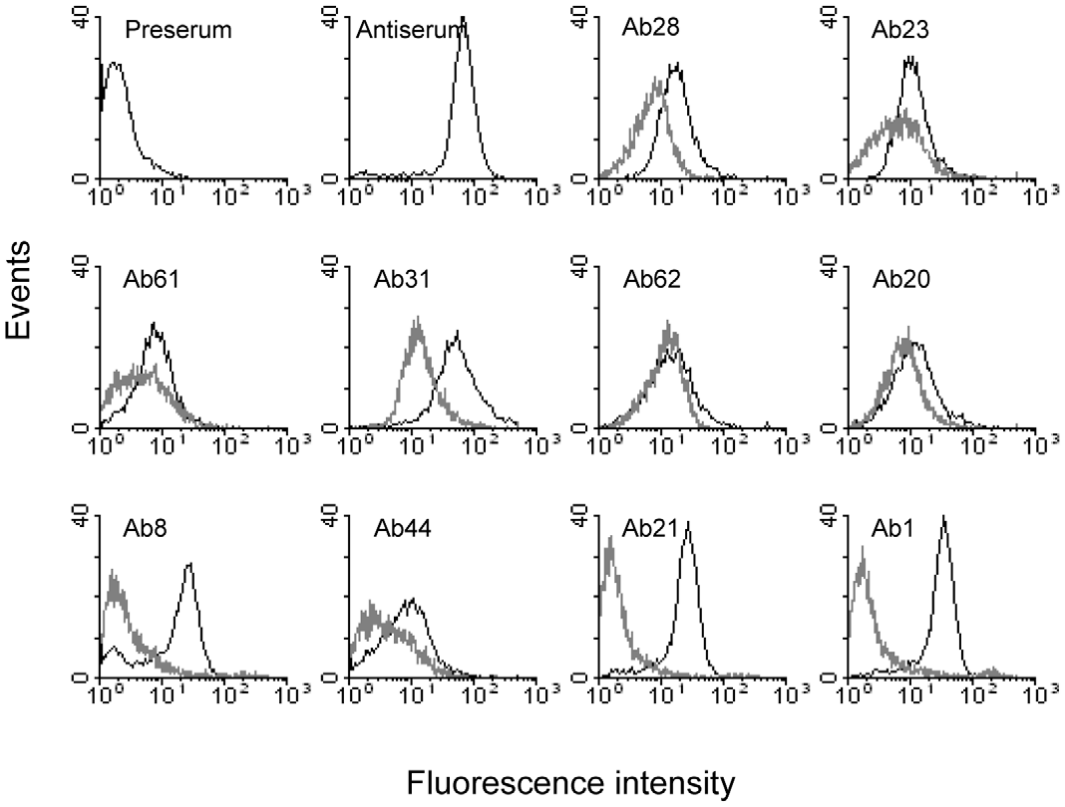
Flow cytometry of affinity-purified antibodies against *S. sanguinis* SK36 whole cells. Affinity-purified antibodies were incubated with (gray line) or without (black line) the protein used for affinity purification, followed by incubation with *S. sanguinis* whole cells. Negative control, pre-immune serum; positive control, anti-pool antiserum used for individual antibody purification. Values are representative of two independent experiments. Values for protein treatments are significantly different from those of protein un-treatments (P<0.05) except for Ab62.

To determine exposure of the pooled proteins on the *S. sanguinis* cell surface, competitive ELISA and Fluorescence-Activated Cell Sorting (FACS) analyses were carried out using affinity-purified antibodies, antigens and bacterial whole cells. For competitive ELISA analysis, each affinity-purified antibody was incubated in the presence or absence of its cognate purified antigen, followed by addition to microtiter wells coated with whole *S. sanguinis* cells (Table 2). The results indicated that all 10 affinity-purified antibodies had reactivity to *S. sanguinis* SK36 whole cells, but, as expected, had less reactivity than whole antiserum. Whole-cell ELISA titers of 9 purified antibodies pretreated with their antigen proteins exhibited significantly reduced activity when compared to untreated purified antibody, especially antibodies against proteins 1, 8 21 and 31 (Table 2). FACS analysis of *S. sanguinis* SK36 whole cells was also performed with affinity-purified antibodies, in the presence and absence of cognate antigens (See Materials and Methods). All 10 purified antibodies produced greater signal intensities than pre-immune serum; FACS values of all purified antibodies except for 62 markedly decreased after pretreatment with specific antigens, especially those of Ab1, Ab8, Ab21 and Ab31 (Fig. 3). This result was consistent with the competitive ELISA (Fig. 2). Thus, both competitive ELISA and FACS suggested that at least 9 of 10 selected proteins were exposed on the *S. sanguinis* SK36 cell surface.

**Table 2:** ELISA titers of affinity-purified antibodies against *S. sanguinis* SK36 whole cells with and without pretreatment with specific antigens.^a^

Serum or antibody	Untreated (titer)	Plus protein (titer)	Inhibition (%)
Preserum	27	-	-
Anti-sham	53	-	-
Antiserum	54613	-	-
Ab28	1024	512*	50
Ab23	512	256*	50
Ab61	512	256*	50
Ab31	3413	427*	88
Ab62	682	512	25
Ab20	512	213*	58
Ab8	32768	512*	98
Ab44	1024	427*	58
Ab21	6827	512*	93
Ab1	13653	512*	96

a Two-fold serial dilutions of affinity-purified antibody was incubated with or without 2 µg specific antigen for 1h and the ELISA titer against *S. sanguinis* whole cells coated in microtiter plate wells were determined. Assays were performed in triplicate and statistically significant differences between protein-treated and -untreated were indicated (*, P<0.05). -, not determined.

### Opsonization activities of affinity-purified antibodies

**Figure 4:**
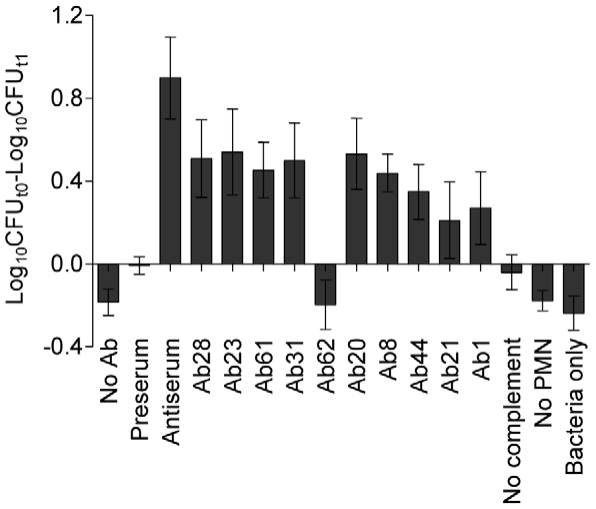
Opsonophagocytic activity of affinity-purified antibodies. *S. sanguinis* cells were incubated with human PMNs, rabbit complement and purified antibody for 1 hr (see Materials and Methods). Log_10_ of bacterial CFU reduction between time 0 and time 1 hr are shown. Controls include pre-immune serum, without complement, without PMNs, or bacteria only. Mean values and standard deviations of at least triplicate assays are shown. Values for purified antibodies are significantly different from the controls (P<0.05) except Ab62.

The specific phagocytic activity in the presence of each affinity purified antibody was analyzed *in vitro* as described [37]. Human polymorphonuclear leukocytes (PMNs) was purified from buffy coat blood and mixed with a fresh culture of *S. sanguinis* SK36. The same amount of each purified antibody based on protein concentrations was used in the assay. The original pre-immune serum and antiserum were used as negative and positive controls, respectively. Reactions without PMNs, without rabbit complement or without antisera were also examined for background. After incubation for 0 or 1 hr, bacterial cells were plated on BHI plates to determine bacterial killing. The bacteria were efficiently phagocytized in the presence of the purified antibodies. Nine of ten purified antibodies exhibited significant enhancement of *S. sanguinis* SK36 phagocytosis by PMNs compared to pre-immune serum or bacteria alone (Fig. 4). In addition, the phagocytic activity in pool-immunized serum was higher than those of individual purified antibodies at the same protein concentrations. The result indicated the possibility of synergistic activity of the antibodies.

## Discussion

Since reverse vaccinology was first developed, there have been several successful applications of this technology to identify vaccine candidates against bacterial pathogens [2], [4]–[11]. In these cases, however, vaccine candidates were eventually obtained through screening individually selected proteins in animal immunization and protection tests. When an infection animal model is not available (such as for emerging pathogens or for dangerous pathogens with high virulence) or the infection animal model is too complicated to screen a large number of candidates, vaccine candidate discovery may be delayed. It also should be noted that a genome-wide screen for vaccine candidates requires a large number of animals and significant time and expense. To solve the problem, we sought to improve this technique by using a pooling strategy in which candidate antigens were combined for immunization and applied comprehensive immunological assays to testing the possible correlates of protection without an infection animal model. This approach will not only decrease the experimental animal usage and reduce time cost for vaccine candidate identification, but also provide a potential assay for synergistic activity between antigens.

We pooled 10 selected proteins from *S. sanguinis* SK36 to immunize rabbits. After pooled immunization, all antigens in the pool elicited immune responses, most of which were high-titer, suggesting that the pool size did not limit immunogenicity (Fig. 1). The antigen-specific fractions of the antiserum were successfully purified by affinity chromatography (Fig. 2). Our result indicated that 9 of 10 affinity-purified antibodies had reactivity to their specific antigens on the *S. sanguinis* SK36 cell surface either by competitive whole-cell ELISA assay or by FACS (Table 2 and Fig. 3). These antibodies also promoted opsonophagocytosis of *S. sanguinis* by human PMNs. Opsonophagocytosis is one of the mechanisms of host defense against pathogen attack. This result suggested the protective potential of each candidate could be assessed via the opsonophagocytosis analysis. Therefore, our approach will rapidly evaluate the immunogenicity and feasibility of vaccine candidates prior to using an infection animal model.

Our results showed the reactivity to *S. sanguinis* whole cells of unfractionated antiserum from pooled-protein immunization was higher than that of individual antibodies purified from the same antiserum (Figs. 3–4 and Table 2). Increased protection has been also reported using mixed proteins as compared to any single protein in *Leptospira kirschneri* [38], [39] and *S. agalactiae* [5]. In this pooled-protein immunization approach, we not only evaluated the potential protection of each antigen, but also obtained information on synergistic activity of the antibodies that will be useful for more powerful vaccine development from multiple antigens. Furthermore, due to the antigenic variability of the different strains within a pathogen, multivalent vaccine approach may be the choice to overcome the antigenic diversity [40]. It is possible to apply our antigen-pooled immunization approach with multiple conserved proteins to examine different strains of a pathogen to find the best combination of multivalent vaccine candidates.

It should be noted that His tag may generate cross-reactive antibodies and then affect the evaluation of pooled proteins because each protein contains the His tag. This interference can be reduced by prior incubation the antisera with the His tag although our result showed the reactivity of the antisera to the His tag was significantly lower than to each protein antigen (Fig. 1C). There seems some cross-reactivity in the affinity purified antibodies (Fig. 2), but the results from the competitive whole-cell ELISA and FACS (Table 2 and Fig. 3) showed this cross-reactivity did not influence the identification of antigenicity and cell surface exposure of the candidates. This cross-reactivity may be caused by the His tag, the limits of affinity purification, or similar protein epitopes [41].

Surface-exposed proteins are regarded as pivotal players in the infectious process of bacterial pathogens [42]. Two classes of surface-exposed proteins, Lpp and CWA proteins, are quite frequently found in reported vaccine candidates in streptococci, including *S. pneumoniae*, *S. agalactiae*, *S. pyogenes* and *S. parasanguinis* [4], [5], [16]–[27]. There may be two reasons for these findings. First, Lpp and CWA proteins are common virulence factors [43]. The functions performed by these proteins may be susceptible to neutralization [44]. Second, Lpp and CWA proteins may be surface-exposed naturally or due to their association with surface structures [45] whereas most membrane proteins would be predicted to be buried within the cell wall in streptococci. Here, we identified 60 Lpp and 38 CWA proteins in the *S. sanguinis* SK36 genome by bioinformatics based on signal peptides, LPXTG motif, and transmembrane helices. Many of them were homologs of vaccines (Table 1). Interestingly, the eight tested Lpp and CWA induced greater reactivity than the two other proteins (numbers 61 and 62) without the structure of Lpp and CWA, implying. that Lpp and CWA proteins are more immunogenic. The high percentage of confirmed surface proteins suggests the feasibility of predicting vaccinogens by bioinformatics, which may also apply to other emerging pathogens.

Five of the 10 candidates (1, 8, 20, 23 and 28) apparently belong to ABC transport systems [28]. ABC transporters are involved in uptake for metal ions, heme, amino acids, peptides, sugars, phosphate, and other nutrients in bacteria. In *S. pyogenes*, five ABC transporters of 16 Lpp have been shown to elicit protection in mice blood against bacterial growth [16]. In *S. pneumoniae*, four ABC transporters (PiuA, PiaA, PsaA and PotD) have been shown to elicit protection in mice against *S. pneumoniae* disease [46]–[48]. FimA, which has been shown in our previous work to protect vaccinated rats from endocarditis caused by different viridans streptococci, is also an ABC transporter [25]. PiuA, PiaA, PsaA and FimA are associated with not only iron, manganese, or zinc ions uptake, but also virulence [46]–[49]. Protein 1 (AdcA), a Zn ABC transporter, was demonstrated to be involved in endocarditis virulence and biofilm formation in one previous study [50], although an independently isolated mutant lacking the same protein retained virulence [28]. We recently found *S. sanguinis* SK36 SsaB, a homolog of FimA in *S. parasanguinis*, is a virulence determinant in the rabbit endocarditis model [28]. Our results and previous findings by others suggest that ABC transporters, particularly those involved in metal ion uptake, are likely candidates for vaccines against diseases caused by streptococci. Protein 8 is a homolog of the PmpA protein of *Borrelia burgdorferi* which was shown to be highly antigenic and contribute to the genesis of Lyme arthritis [51], [52]. Protein 21, PrsA, is an ortholog of a peptidyl-prolyl isomerase. PpmA (i.e. PrsA) of *S. pneumoniae* that has been associated with pneumococcal virulence [53]. Mutation of *prtM* (*prsA*-like) attenuated the virulence of *Streptococcus equi* in a mouse model [54]. CWA protein 31 (CbpA) is a collagen-binding protein suggested to be involved in platelet aggregation [55]. Platelet aggregation is thought to play an important role in causing bacterial endocarditis [55]–[58]. The mutation of CbpA in *S. sanguinis* substantially inhibits its platelet aggregation compared with the wild-type [59]. The data on homologs of proteins 1, 8, 21 and 31 in previous studies suggest these proteins may be involved in virulence and have the potential to become vaccine candidates. Interestingly, the antibodies affinity-purified by these four proteins demonstrated stronger reactivity to the cell surface of *S. sanguinis* than other tested Lpp and CWA proteins based on the consistent results from both whole-cell ELISA and FACS ( Table 2 and Fig. 3). Among the 8 tested Lpp and CWA proteins, nevertheless, there was no significant difference in the opsonophagocytic activities of their affinity-purified antibodies at equal concentrations.

The two non-Lpp and CWA proteins examined in the pool had different performances on the immunogenicity and opsonophagocytic activity. With a signal peptide and no transmembrane domain, protein 61 is probably a secreted protein. It is predicted as a carbamate kinase. Carbamate kinase has been shown to be a secreted or cell wall-associated protein in *S. pyogenes* [60], *Salmonella enterica* [61] and *Trichomonas vaginalis* [62]. Carbamate kinase is one of the three components of arginine-deiminase pathway identified as a cell wall associated enzyme system in *S. pyogenes* [60]. This pathway is absent in the majority eukaryotes including humans and has been suggested as a vaccine candidate or novel drug target. Here, protein 61 elicited an immune response in rabbits (Fig.1C) that was reactive with whole cells (Table 2 and Fig. 3) and which enhanced opsonophagocytic activity (Fig. 4). Protein 62, a putative sugar-binding protein, is a surface–associated protein but not Lpp and CWA protein based on its signal peptide and a transmembrane domain. It possesses orthologs in *S. gordonii* and *S. mutans* but not in *S. pyogenens*, *S. pneumoniae*, *S. agalactiae* or *S. thermophilus*. To date, there is no report on the potential of this protein to be a vaccine candidate in bacteria. Our results showed protein 62 elicited immune responses but lower than those of Lpp and CWA proteins in pool immunization (Fig. 1C). Its antibody failed to be purified from pool-immunization antisera (Fig. 2) because of low titer or an unknown reason, which prevented us from determining its cell-surface exposure or opsonophagocytic activity.

Taken together, most of 10 proteins we selected here were shown to elicit strong immune response and the potential to become vaccine candidates. The results also suggested that our approach to predict vaccine candidates using bioinformatics and examine the immune responses using pooled proteins will facilitate vaccine development against emerging pathogens.

## Materials and Methods

### Animal ethics

Animals were treated humanely and in compliance with all applicable federal guidelines and institutional policies. All of the procedures were approved by Virginia Commonwealth University Institutional Animal Care and Use Committee.

### Bacterial strains, plasmids and primers

*S. sanguinis* strain SK36, whose genome has been sequenced [63], was grown at 37°C in brain heart infusion broth (BHI; Difco Inc., Detroit, MI) supplemented with 1.5% agar as described previously [50]. The strain was cultured under anaerobic conditions. *E. coli* strains NovaBlue and BL21 (DE3) pLysS (Novagen, Gibbstown, NJ) were used as hosts for plasmid production and protein expression, respectively. Vector pET-46 Ek/LIC (Novagen) containing an N-terminal cleavable His•Tag sequence was used in gene cloning and expression. Primers for PCR amplification of selected genes are listed in Table 3. To directly clone PCR fragments into pET-46 Ek/LIC vector without ligation, each forward primer and reverse primer contained 5′-end sequence tails as described by the manufacturer.

**Table 3:** Properties and primer sequences for *S. sanguinis* pool-protein genes.

ID^a^	SSA#	Putative function	Primers	Sequences (5′→3′)	Position^b^	MW^c^
28	SSA_2352	ABC-type nitrate/sulfonate/bicarbonate transporter	28F	GACGACGACAAGATCAAAAAAAGTTATAAAGTCTTGTTGGCAGGT	4∼999	39.1
			28R	GAGGAGAAGCCCGGTTTATTTTATATAATCGTTGCTAAATCCCTT		
23	SSA_0074	ABC transporter substrate-binding protein-sugar transport	23F	GACGACGACAAGATACCAAGTGGGGAAAATAGTAAAGGAAA	73∼1344	49.2
			23R	GAGGAGAAGCCCGGTTTATTTCACATCAACTGCAACCGTTTC		
61	SSA_0739	Carbamate kinase	61F	GACGACGACAAGATAGCAAATCGTAAAATCGTTGTAGCCTT	4∼945	35.4
			61R	GAGGAGAAGCCCGGTTTAGCCTTTTTCAATAATCGTGCCGC		
31	SSA_1663	Collagen-binding protein A	31F	GACGACGACAAGATCCAAGCAGCTGAGTTTGGTGATGTT	91∼4452	158.7
			31R	GAGGAGAAGCCCGGTTTAAGAACTTTCTTCGAGTTCCCCTTA		
62	SSA_0218	Sugar-binding periplasmic protein	62F	GACGACGACAAGATCGAAAAAGTCCTGCGTATCGGGGTC	91∼1281	51.8
			62R	GAGGAGAAGCCCGGTTTATTTTCCGCTTTCTATTTCTCTTTG		
20	SSA_0893	ATP-binding cassette transporter-like protein	20F	GACGACGACAAGATCAGACAGATCAATCAACAACAGGCCA	94∼1263	44.6
			20F	GAGGAGAAGCCCGGTTTAAGCTTTAACATGCTGTCCTACCT		
8	SSA_1038	Lipoprotein	8F	GACGACGACAAGATCAACAAAAAACAATGGCTAGGTCTTGG	4∼1053	38.7
			8R	GAGGAGAAGCCCGGTTTATTTGTCAGGAACAGTGATGCTGC		
44	SSA_0146	Possible ATPase involved in DNA repair, contains gram positive anchor	44F	GACGACGACAAGATAGCAGATGAGGAGCAGCCTGTTGC	106∼2373	82.7
			44R	GAGGAGAAGCCCGGTTTAATCTTCCTTTTTTCTACTACGGAG		
21	SSA_0753	Foldase protein prsA precursor	21F	GACGACGACAAGATCAAGAAAAAAATATTTGCAGGAGCAGTG	4∼1005	38.2
			21R	GAGGAGAAGCCCGGTTTATTCTGCGGCTGAGGATGAAGC		
1	SSA_0138	Metal-binding (Zn) permease	1F	GACGACGACAAGATCAAAAAAATTAGCTTACTATTAGCAGGTC	4∼1500	58.1
			1R	GAGGAGAAGCCCGGTTTAGTGCGCCAACATCTCTTGGGC		

a identifying number.

b fragment position from 5′-end to 3′-end in ORF.

c molecular weight of protein including His-tag.

### Genomic analysis and surface protein prediction

To predict surface-exposed proteins, all ORFs in our annotation of *S. sanguinis* genome were analyzed. The SignalP 3.0 program [14] was used with the gram-positive setting to predict signal peptides within 70 residues of the N-terminus of each ORF. The TMHMM program [15] was used to identify transmembrane proteins and locate hydrophobic domains. To identify lipoproteins, LipoP 1.0 [64] was used [28], [64]. To recognize cell-wall anchored proteins, the fuzzpro program in the EMBOSS package [65] was used to identify the C-terminal signal containing the LPXTG pattern [29].

Comparative genomic analyses were carried out to identify conserved proteins in *S. sanguinis* using BLASTP as previously described [13]. *S. sanguinis* proteins were compared to completed streptococcal genomes downloaded from the NCBI protein database. Significant matches (E<1e^−5^) were analyzed to find putative proteins with homologs in at least two other streptococcal species. To evaluate the accuracy of computational prediction, we collected all literature-reported leading vaccine candidates in streptococci and downloaded their protein sequences from NCBI databases. The sequences were compared with *S. sanguinis* proteins to identify vaccine homologs.

### Gene cloning and Protein purification

Predicted Lpp and CWA ORFs were selected for PCR amplification. Because proteins with a high number of transmembrane domains are difficult to express, the regions encoding the transmembrane domains predicted by TMHMM program in ORFs were excluded. To directly clone the candidate genes, PCR was performed using the specific pET Ek/LIC primers, *S. sanguinis* SK36 genomic DNA template, and Platinum Taq DNA Polymerase High Fidelity (Invitrogen, Carlsbad, CA). PCR amplification was conducted as follows: 94°C for 1 min to denature template; 30 cycles of 94°C for 30 s, 60°C for 30 s and 68°C for 1 min per kb of amplicon. The amplicons were purified with a PCR purification kit (Qiagen, Valencia, CA), and mixed with pET-46 Ek/LIC vector for transformation of NovaBlue competent cells as described by the manufacturer. After the recombinant plasmids were confirmed to have the expected inserts by colony PCR and sequencing, they were re-transformed into *E. coli* BL21(DE3)pLysS for protein expression and purification.

BL21(DE3)pLysS cells containing the appropriate plasmid were cultured overnight in Luria-Bertani (LB) medium supplemented with 100 µg/ml ampicillin at 30°C with shaking. The culture was diluted 100-fold with fresh LB medium the next morning and incubation continued until the OD_600_ reached approximately 0.5. After addition of isopropyl β-D-1-thiogalactopyranoside (IPTG) to 0.1 mM to induce protein expression, the culture was incubated with vigorous shaking at 20°C overnight (16h). Bacterial cells were harvested by centrifugation and stored at −80°C. Lysis of the cell pellets was performed at room temperature using BugBuster buffer containing 25 U/mL Benzonase® Nuclease (Novagen). After centrifugation to remove debris at 16,000×g for 20 min at 4°C, the supernatant was collected for protein purification. Protein was purified using a Novagen His•Bind® Column Chromatography kit as described in the manufacturer's protocol. The purified protein was dialyzed against 10 mM Tris-Cl, pH 8.0 buffer (containing 20 mM NaCl, 0.1% glycine) overnight. The protein was concentrated using an Amicon® Ultra centrifugal filter (Millipore, Billerica, MA).

### Pooled-protein vaccination

Ten purified proteins were pooled for rabbit immunization. The protocol received Institutional Animal Care and Use Committee approval and complied with all applicable federal guidelines and institutional policies. The pool containing equimolar amounts of ten purified proteins (total 1 mg) was mixed 1∶1 (v/v) with complete Freund's adjuvant (CFA) (Difco Laboratories, Sparks MD) and injected subcutaneously into 3 specific-pathogen free New Zealand White rabbits from which pre-immune serum had been collected. For a sham vaccination control, one milligram of His-tagged *P. gingivalis* Kgp protein described previously [25], also mixed 1∶1 (v/v) CFA, was used. Three weeks after initial immunization, blood was collected and rabbits were boosted with the identical proteins mixed 1∶1 (v/v) with incomplete Freund's adjuvant. Three weeks later, antisera were collected for further analysis.

### Western blot

To examine the reactivity of the antisera to each protein used for immunization, a Western blot was performed as described [66]. Briefly, ten His-tag purified proteins (10 ng each/well) were separated on a 7.5% SDS-PAGE gel and transferred to a nitrocellulose membrane. The transferred membrane was blocked with TTBS (Tris-buffered saline and 0.1% Tween 20) and 1% BSA for 1h, and incubated at room temperature for 1 h with 1∶2000 diluted antiserum. After washing with TTBS, the membrane was incubated with 1∶4000 Goat anti-rabbit IgG–AP conjugates (Promega, Madison, WI) at room temperature for 1 h. The color reaction of the membrane was developed with Western Blue Stabilized Substrate for Alkaline Phosphatase (Promega).

### ELISA assays

Indirect ELISA was applied to detect the titer of the antisera against specific candidate proteins. To determine the optimal concentrations of the reagents for ELISA, checkerboard titrations (CBT) were performed as described [67]. Ten proteins were optimized by criss-cross serial dilution analysis using antiserum. Pre-immune serum was used to estimate the background. After criss-cross serial dilution determining the optimal concentration of antigen proteins for indirect ELISA, Immulon 2 HB microtiter plates (Nunc, Rochester, NY) were coated with 100 µl purified protein overnight at 4°C. After incubation with blocking buffer and three washes with ddH_2_O, two-fold serial dilutions of 100 µl antiserum (or pre-immune serum) were added and the plates were incubated at room temperature for 2 h. After blocking and washing again, a 4000-fold dilution of goat-anti-rabbit IgG–AP conjugate (Promega) was added. After 2 h incubation at room temperature, the color reactions were developed with *p*-nitrophenyl phosphate (Sigma, St. Louis, MO) and the absorbance was measured at 405 nm. Titers of protein-specific antibodies were defined as the dilution that gave an OD_405_ value 0.1 higher than the pre-immune serum background.

For whole-cell ELISA, 10^6^ colony-forming units (CFU) of *S. sanguinis* SK36 cells harvested at early stationary phase were coated onto plate wells according to a method described previously [36]. The antiserum was added and allowed to interact with bacterial surface proteins. After removing the free antiserum by five washes with 200 ul PBST each, the bound antiserum was quantified by adding goat-anti-rabbit IgG–AP, as with the indirect ELISA.

For competitive ELISA, affinity-purified antibodies were 2-fold serially diluted in 96-well untreated polystyrene microtiter plates (Nunc, Thermo Scientific, Waltham, MA). Two sets of serially diluted samples for each purified antibody were prepared. One diluted sample was mixed with its specific antigen at a final concentration of 10 µg/ml in each well. The other diluted samples were mixed with BSA only. Mixtures were incubated at 37°C for 1 h with gently shaking. After incubation, the mixtures were transferred to the *S. sanguinis* SK36 cell-coated plate wells and incubated at 37°C for 1 h. After removing the free antibodies by five washes with 200 ul PBST, the bound antibodies were detected by indirect ELISA, as described above.

### Antiserum affinity purification

To purify antigen-specific antibody from the pool-immunized antiserum, affinity chromatographic purification was performed using AminoLink Plus Column kits (Pierce Protein Research Products, Rockford, IL) according to the manufacturer's protocol. Briefly, the purified proteins without Tris were first covalently linked on solid AminoLink Plus Columns with sodium cyanoborohydride solution. After blocking the remaining active sites of the column with Quenching Buffer and washing away reactants and non-coupled protein, the antiserum was loaded on the column. After four washes with Coupling Buffer (Pierce Chemical Company), the affinity purified antibody was eluted using ImmunoPure Gentle Ag/antibody buffer (Pierce Protein Research Products). The elution fractions of interest were pooled by checking absorbance at 280 nm. After dialysis, the purified immunoglobulins were concentrated with Amicon® Ultra centrifugal filters (Millipore). The immunoglobulin concentrations were determined by OD_280_ and equivalently diluted. Serial dilutions of the purified immunoglobulins were assayed using ELISA to examine their purity and specificity.

### FACS analysis

FACS analysis was carried out as described previously [5]. Briefly, *S. sanguinis* SK36 were grown in Todd-Hewitt broth (Difco Inc.) to an OD_660_ of 0.6. Bacteria were collected by centrifugation, washed twice with PBS and incubated with newborn calf serum (Sigma) for 20 min at room temperature. The harvested cells were then incubated at 4°C for 1 h in 200 µl of pre-immune sera, immune sera, or affinity-purified antibody pretreated with or without its specific antigen (to final concentration 10 µg/ml) for 1 h at 4°C. After centrifugation and washing with 0.1% BSA in PBS, cells were incubated at 4°C for 1 h with 50 µl of R-phycoerythrin-conjugated F(ab)2 goat anti-rabbit IgG (Pierce Protein Research Products) diluted 1∶100 in PBS containing 0.1% BSA and 20% newborn calf serum. Bacteria were washed with 0.1% BSA in PBS, resuspended in 200 µl PBS and analyzed using a flow cytometer (Guava EasyCyte Mini System, Guava Technologies, Hayward, CA). FACS data were analyzed by the program Win MDI 2.9.

### Opsonophagocytosis

Specific phagocytic activity in the presence of each affinity purified antibody was measured in vitro as described [37]. Human PMNs were isolated from buffy coat blood (Virginia Blood Service) using sedimentation through a solution of hydroxyethyl starch followed by density centrifugation over Histopaque (Sigma) according to the manufacturer's protocol. Phagocytosis was examined in a reaction of 125 µl containing ∼2×10^6^ human PMNs, ∼7×10^6^ CFU of *S. sanguinis* SK36 cells, 10% rabbit complement (Sigma), and affinity-purified antibody with shaking at 250 rpm at 37°C for 1 h. After 0 or 1 h of incubation, the mixture was diluted in sterile ddH_2_O and plated on BHI agar plates.

### Statistical analysis

Competitive ELISA and FACS data were analyzed by unpaired Student's test based on their titer and geometric mean fluorescence intensity, respectively. Other data on ELISA and opsonophagocytosis were examined by ANOVA with a Dunnett multiple-comparison post hoc test. P<0.05 represents a statistically significant difference.
